# EHMTI-0009. A particular case of cluster headache

**DOI:** 10.1186/1129-2377-15-S1-C60

**Published:** 2014-09-18

**Authors:** D Tertan, OB Tertan

**Affiliations:** 1 Neurology, Clinical Hospital Pelican, Oradea, Romania; 2 University of Medicine and Pharmacy, Cluj Napoca, Romania

## Introduction

Cluster headache represents a severe, strictly unillateral retroorbital short lasting headache, accompanied by permanent parasimpathetic autonomic features; attacks occur regularly for one week to one year, separated by pain- free periods that last at least one month.

## Aims

We present a case of cluster headache as the presenting symptom leading, in the end, to the diagnosis of Multiple Sclerosis(MS) with a demyelinating lesion in the left trigeminal nucleus and tract.

## Methods

We reviewed the case retrospectively, including the clinical, laboratory and radiological data.

## Results

Our patient, female, aged 31 years, was admitted to our hospital with attacks of headache of short duration 10-20 minutes, that occur 4-5 times a day, usually during sleeping, accompanied by rhinorrhea, eyelid edem, ptosis, miosis and conjunctival injection. The pain is so excruciating that the patient describes it as "suicide headache", followed by an incomplete abolition of the pain after 50-100 mg Sumatriptane per day, 600 mg Magnesium daily and Capsaicin cream. Cerebrospinal fluid revealed mildly elevated protein. Magnetic resonance imaging showed a non-hancing, non restricting lesion within the pontine portion of the left trigeminal nucleus and tract.There were also numerous periventricular and callosal white T2 hyperintensities consistent with demyelination. The diagnosis of MS was confirmed by additional lesions in the cervical spinal cord, presence of oligoclonal bands, intrathecal Ig G synthesis.

## Conclusion

The case suggests that in patients with any primary headache, especially young population, neuroimaging is often useful in order to exclude structural lesions.

No conflict of interest.

